# Phytotoxicity and phytoremediation potential of *Lemna minor* exposed to perfluorooctanoic acid

**DOI:** 10.3389/fpls.2024.1493896

**Published:** 2025-01-27

**Authors:** Azam Noori, Lorena Corbelli, Erin Lincoln, Sara Thomas, Jasmine Jones, Sara L. Nason, Jason C. White, Riley Lewis, Christy L. Haynes

**Affiliations:** ^1^ Department of Natural Sciences, Merrimack College, North Andover, MA, United States; ^2^ Connecticut Agricultural Experimental Station, New Haven, CT, United States; ^3^ Department of Chemistry, University of Minnesota, Minneapolis, MN, United States

**Keywords:** emerging compounds, *Lemna minor*, PFOA, phytoremediation, phytotoxicity

## Abstract

Perfluorooctanoic acid (PFOA) is one of the highly toxic compounds which was phased out of application in consumer products in 2015 due to its harmful effects on human and environmental health. However, this chemical was in use for many years and is still found in water resources. This study focuses on the physiological response of duckweed (*Lemna mino*r) exposed to PFOA so as to determine phytotoxicity and the potential of this aquatic species to remove PFOA from the environment. A time-dependent phytotoxicity assay showed that exposure to 0.1 µg/L PFOA for 14 days resulted in the loss of chlorophyll pigment and 15-25% more chlorosis than in controls. Although exposure to PFOA for seven days resulted in chlorosis, no significant impact on physiological parameters such as photosynthetic pigment or anthocyanin content were detected. The analysis of cellular size on day zero and seven of the experiment showed that the control group showed significantly larger cell size after seven days (213 ± 6.5 µm²) compared with the day zero group (186 ± 18 µm²), while the size of the PFOA exposed group (198 ± 13 µm²) did not change significantly after seven days compared with the day zero group. The nuclear size increased significantly by 13% upon exposure to PFOA compared with the controls (ρ < 0.0001). The concentration of essential elements K, Cu, Fe, Mn, Zn, Mo were reduced in *L. minor* exposed to PFOA compared with the controls by 39.6, 33.4, 42.1, 35.2, 31.9, 40.2%, respectively. Additionally, PFOA accumulated in *L. minor* fronds and roots with an average bioaccumulation factor of 56 ± 7. Overall, while some symptoms of toxicity were observed, this study shows that *L. minor* can tolerate up to 0.1 µg/L PFOA, a commonly found concentrations in water bodies, and can remove PFOA from water. This study provides invaluable information regarding the phototoxicity impacts of PFOA on aquatic species and the potential for aquatic phytoremediation of PFOA.

## Graphical Abstract

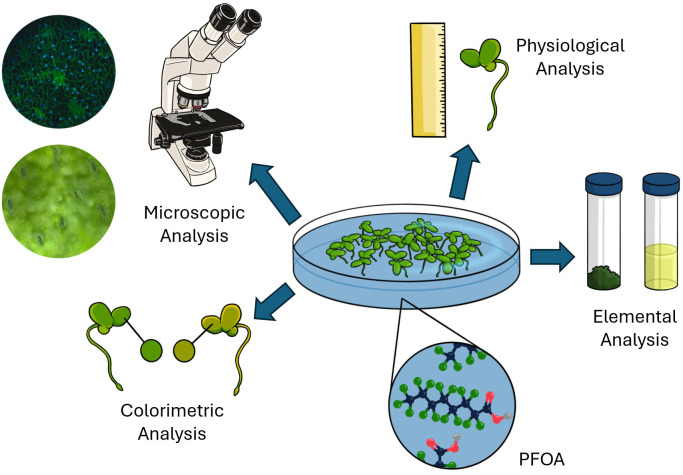

## Introduction

Per- and poly-fluoroalkyl substances (PFAS) are a class of compounds characterized by robust carbon-fluorine (C-F) bonds, which render them resistant to degradation, hydrolysis, and photolysis. Due to their persistence, they are often referred to as “forever” chemicals (Wang et al., 2020; Mancinelli et al., 2022; Wee and Aris, 2023). These substances have been extensively synthesized and employed since the 1940s, finding widespread applications in various industrial and consumer products, such as automobile components, firefighting foams, water-resistant textiles, cleaning agents, nonstick cookware, food packaging, personal care items, and various other commonly used products (Buck et al., 2011; Cousins et al., 2020; Bulson et al., 2023). As a consequence of their extensive use, PFAS have been detected in the atmosphere, soil, and sediment, as well as both surface and groundwater globally, leading to their designation as “everywhere” chemicals (Crone et al., 2019).

PFAS can be classified into distinct subcategories based on their chemical functional groups, including carboxylic and sulfonic acids, sulfonamides, amines, and ether-based compounds (Cousins et al., 2020; Glüge et al., 2020; Su et al., 2024). Notably, many PFAS exhibit hydrophobic and lipophobic characteristics, which are linked to the length of the fluorinated carbon chain portion of the molecule (Gagliano et al., 2020). According to the criteria established by the Organization for Economic Cooperation and Development (OECD) and the United Nations Environmental Program, PFAS with six or more carbon atoms are categorized as long-chain compounds (OECD/UNEP Global PFC Group, 2013). Perfluorooctanoic acid (PFOA), which is among the top five PFAS compounds of concern, has 8 carbons and is a long-chain PFAS with an approximate half-life of 3.5 years in human blood (Perez et al., 2023; Rosato et al., 2024).

Given the worldwide presence of PFAS, the health impacts of exposure to these chemicals, and the difficulty in removing them from the environment, the development of novel and effective strategies to mitigate their concentration in the environment is needed. Various chemical and physical methods such as chemical oxidation (Taher et al., 2024), treatment with granular activated carbon (McNamara et al., 2018; Herkert et al., 2020), carbon-based sorbents (Bui et al., 2024), or electrochemical degradation (Sharma et al., 2022) have been used for the remediation of PFAS. However, these methods are not effective for all types of PFAS in the various environmental compartments (Zhou et al., 2024). Biological methods such as phytoremediation may offer significant potential for reducing PFAS levels in the environment (Li J. et al., 2022; Pi et al., 2017; Zhang et al., 2019). Previous studies have reported the accumulation of PFAS in plant tissues (Piva et al., 2023; Zhang et al., 2023). Greger and Landberg (2024) investigated the responses of 17 wetland species to PFAS exposure in a highly contaminated lake and showed that plants such as *Eriophorum angustifolium* and *Carex rostrata* had a high potential for phytoremediation of these chemicals. Qian et al. (2023) studied the translocation of four classes of PFAS by five different fern species. The authors reported that bioaccumulation of PFAS in plants varies based on both plant and PFAS properties, such as root length and surface area, as well as molecular size and solubility of PFAS. Most studies have focused on phytoremediation approaches in plants grown in soil or emergent species in surface water. However, considering the common detection of PFAS in surface waters, it is essential to study the impact of PFAS on a more diverse range of plants, such as submerged species, and to explore their phytoremediation potential.

Determining the phytotoxic responses of a range of plant species to the contaminant of concern is an important first step to selecting a phytoremediation species. The ideal plant species for this purpose should tolerate the target contaminant with minimal toxicity responses while effectively accumulating the contaminant from the environment. Although the toxicity responses of plants to PFAS are generally low, studies indicate that plant photosynthetic rates and growth can be negatively impacted by exposure to these contaminants (Karamat et al., 2024; Li et al., 2022). Duckweed (*Lemna minor* L.) is a submerged species commonly used as a model for assessing phytotoxicity responses due to its rapid growth and widespread distribution (Bal Krishna and Polprasert, 2008; Ali et al., 2020). The current study focuses on the impact of perfluorooctanoic acid (PFOA), one of the most commonly detected PFAS in the environment, on the aquatic species *L. minor*. The study aims to assess this species phytotoxicity responses, bioaccumulation potential, and capability to decrease the concentration of PFOA in water. This work advances our understanding of novel and sustainable strategies to manage PFAS contamination and minimize risks associated with exposure in the environment.

## Materials and methods

### Experimental setup

*Lemna minor* obtained from Carolina biological (cat. no. 161820) were exposed to aqueous PFOA (Sigma Aldrich cat. no.33824); plants grown in chlorine-free commercially available Poland spring water with the pH of 7.2, were used as the control group. No additional nutrients were added to avoid any possible interaction with PFOA. Plants were grown in polypropylene containers to eliminate PFOA adherence to the surface of the container. Polypropylene flasks are reported to have minimal to no absorption of PFAS (Cao and Xiao, 2024). Each 6x6 cm container was filled with 100 mL water that was or was not amended with PFOA. *Lemna minor* with an approximate mass of 3 g were weighed and added to each container. Experiments were conducted in a greenhouse under controlled conditions at 25 ± 2°C and 16/8 hrs day/night under two fluorescent HO bulbs (HydroplanetTM, 6500 K lumens with an output of 54 watts). A minimum of three replicates per treatment was used for each assay, and fresh plants were utilized for every assay to ensure consistency and reliability in the results.

### Half-maximal inhibitory concentration (IC_50_) assay

**Figure 1:**
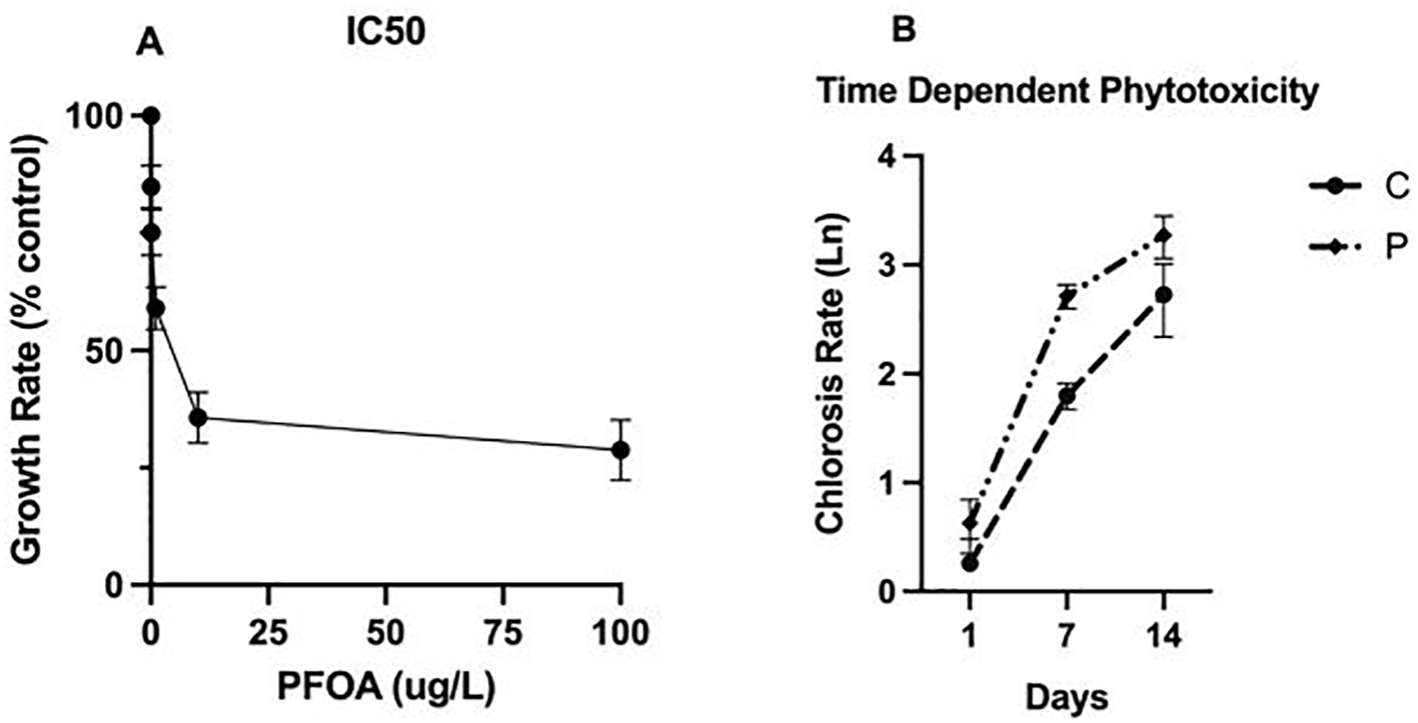
**(A)** The Half-Maximal Inhibitory Concentration (IC50) assay in *L. minor* exposed to PFOA concentrations ranging from 0 to 100 µg/L for seven days. The growth rate results are presented as a % control. **(B)** The time-dependent phytotoxicity impacts resulting from exposure to PFOA. Chlorosis surfaces in *L. minor*, exposed to concentrations of (C) 0 and (P) 0.1 µg/L PFOA for 1, 7, and 14 days, were analyzed using ImageJ. The values, based on three replicates per exposure and time point, are presented in logarithmic scale.

To determine the IC*_50_* of PFOA, the growth rate of *L. minor* was recorded upon exposure to PFOA at 0, 0.01, 0.1, 1, 10 or 100 µg/L for seven days. These PFOA concentrations were selected based on the common detected levels in water bodies (Environmental Working Group, 2022). The number of fronds on the first day and after seven days of exposure were used to determine the impact of PFOA on plant growth using ImageJ (Schneider et al., 2012). A minimum of three replicates was used per exposure. The concentration of PFOA that resulted in a 50% growth compared with the control group was 0.1 µg/L (**Figure 1A**) and was used as the dose for the remainder of the experiments.

### Time-dependent phytotoxicity

Plants were exposed to 0 or 0.1 µg/L PFOA for 14 days. Images of at least three biological containers per group were recorded on days 1, 7, and 14 and analyzed using ImageJ (Schneider et al., 2012). The loss of chlorophyll pigment and the presence of white or yellow coloration in the leaves were used as an indication of chlorosis. The data were calculated using the following equation and reported as chlorosis percentage.


$$Chlorosis\;Index\;(\%)=\frac{Depigmented\;Area\;(mm^{2})}{Total\;Leaf\;Area\;(mm^{2})}x\;100$$

Based on the results, exposure to PFOA for seven days showed less chlorosis than the 14-day exposure (**Figure 1B**). Therefore, seven days exposure was considered as the experimental period to assess phytoremediation potential in the remainder of the experiments.

### Physiological analysis

To assess the impact of PFOA on photosynthetic pigments, chlorophyll a (Chla), chlorophyll b (Chlb), and carotenoids were extracted from *L. minor* exposed to 0.1 µg/L PFOA for seven days according to Lichtenthaler (1987) with minor adjustments. In brief, 1 gram of freshly harvested *L. minor* was homogenized with 3 mL of 95% (v/v) ethanol in water after the removal of excess water from the surface of the plant by brief air drying in a strainer. Subsequently, the homogenized samples were transferred to 15 mL tubes, and the volume was adjusted to 10 mL using 95% ethanol. The content of each tube was mixed using a vortex. Following this, the tubes were incubated at 4°C in the dark for 24 hours, followed by centrifugation at 12,000 g at 4°C for 10 minutes. The resulting supernatant was used to detect photosynthetic pigments using a spectrophotometer (Evolution 201 UV visible spectrophotometer, Thermo Scientific, Waltham, MA) at 470, 648.6, and 664.2 nm. The quantities of Chl a, Chl b, and carotenoids were computed using the following equations:


$$Chla=13.36\;x\;A_{664.2\;}-\;5.19\;x\;A_{648.6\;}$$

$$Chlb=27.43\;x\;A_{\begin{array}{c} 648.6\;\; \end{array}}-\;8.12\;x\;A_{664.2}$$

$$C_{x+c}\;=\;\frac{\left(1000\;x\;A_{470}-\;2.05\;x\;Chla\;-\;114.8\;x\;Chlb\;\right)}{245}\;$$

Where A_470_, A_648_, and A_664.2_ are the absorbance values at the respective wavelengths. The calculated values for Chla, Chlb, and carotenoids were normalized to the fresh weight of *L. minor* and expressed as concentrations per g FW/mL.

Anthocyanin levels were assessed to determine the stress response in *L. minor* exposed to PFOA according to Nakata et al. (2013). In brief, 200 mg of *L. minor* underwent cryogenic grinding, followed by homogenization with 1 mL of a solution composed of 45% methanol and 5% acetic acid in water (v/v). The resultant homogenate was then incubated in darkness at 4°C for two hours, followed by centrifugation at 12,000 g at 4°C for 5 minutes. Subsequently, the absorbance of the supernatant was measured spectrophotometrically at 530 and 637 nm. Standard solutions of cyanidin 3-glucoside, with serial dilutions, were used for calibration, and anthocyanin concentration in g FW was computed using the equation below:


$$Anthocyanin\;Conc.\;(gFW)\;=\;\frac{A_{530\;}-A_{637}}{\epsilon \;x\;L}x\;DF$$

Where A_530_ and A_637_ represent the absorbance values at 530 nm and 637 nm, respectively, *ϵ* is the molar absorptivity of cyanidin 3-glucoside, *L* is the path length (1 cm), and *DF* is the dilution factor.

### Oxidative stress indicators

To assess oxidative stress resulting from PFOA exposure, concentrations of hydrogen peroxide (H_2_O_2_) and malondialdehyde (MDA) were determined in the plant tissues. The H_2_O_2_ content in *L. minor* was quantified according to Velikova et al. (2000). A 0.1% (w/v) cold trichloroacetic acid (TCA) solution dissolved in dH_2_O served as the extraction buffer. The colorimetric solution comprised a potassium phosphate buffer (10 mM, pH 7) and freshly prepared KI in a 10 mM potassium phosphate buffer. In brief, 200 mg of *L. minor* underwent cryogenic grinding, followed by homogenization with the combined extraction and colorimetric solution (TCA: KI: K phosphate buffer) at a ratio of 1:2:1 for 10 minutes at 4°C. The total volume of the solution was 10 times that of the plant mass (1 mL for 100 mg). Negative control samples were prepared with deionized water (dH_2_O) instead of KI. After centrifugation at 12,000 g for 15 minutes at 4°C, the supernatants were incubated at room temperature for 20 minutes and subsequently analyzed spectrophotometrically at 350 nm. All samples and standard solutions for H_2_O_2_ were shielded from light by preparing them in amber tubes. The extinction coefficient for H_2_O_2_ at 350 nm was determined using standard solutions for H_2_O_2_. Data analysis was conducted using the Beer-Lambert equation, and results were reported as millimoles per gram of fresh weight (mmol/gFW).

If exposure to PFOA induces lipid peroxidation in *L. minor*, it will lead to the release of malondialdehyde (MDA) as a byproduct. Elevated MDA levels serve as a biomarker of oxidative stress, indicating cellular membrane damage caused by PFOA exposure. Lipid peroxidation was determined using the thiobarbituric acid reactive substances (TBARS) assay to measure MDA levels following Ma et al. (2013) with some modifications. MDA, a byproduct of lipid peroxidation, reacts with trichloroacetic acid (TCA) and thiobarbituric acid (TBA), forming a pink chromophore measurable by spectrophotometry. In brief, 500 mg of fresh *L. minor* were homogenized with 3 mL of 0.1% (w/v) TCA. The resulting extracts underwent centrifugation at 8000 g for 15 min, and the supernatant was transferred to new tubes containing 2 mL of 20% (w/v) TCA and 2 mL of 0.5% (w/v) TBA. After heating at 95˚C for 30 minutes, absorbance was measured at 532 and 600 nm using a UV-vis spectrophotometer (Thermo Scientific, Waltham, MA). The MDA concentration was determined using the Beer-Lambert equation and reported as mM:


$$MDA\;Conc.\;(mM)=\;\frac{A_{532}-\;A_{600}}{MDA\;Extinction\;Coefficient\;x\;Pathlength\;of\;Cuvette}$$

Where the *extinction coefficient* for MDA is 155 mM^-1^ cm^-1^, and the pathlength of the cuvette is 1 cm.

### Stomatal pore size

Stomata play a crucial role in regulating gas exchange and water loss in plants. Understanding the changes in stomatal pore size can provide valuable insight into the potential impacts of PFOA on stomatal function and conductance. Microscopic images of *L. minor* leaves were captured using a light microscope (Zeiss Axiolab 5, Germany). The width of stomatal pores was determined in each exposure group using ImageJ software version 1.54d (Schneider et al., 2012). Statistical analysis was conducted on data obtained from 30 stomata per exposure using SPSS 29.

### Live cell imaging

To assess the impact of PFOA exposure on cellular structure, *L. minor* fronds from control plants (C-7) and those exposed to 0.1 µg/L PFOA for seven days (P-7) were used for live cell imaging employing a fluorescent staining method adapted from Schoor et al. (2015). Briefly, freshly harvested *L. minor* fronds were introduced to the stabilizing buffer for 5 minutes. Subsequently, the samples were transferred to a clean dish containing either Calcofluor white or DAPI fluorescent dye, as detailed in **Supplementary Table S1**, and incubated in darkness for 15 minutes. Afterward, the samples underwent two successive washes with the buffer, each lasting 10 minutes. The prepared samples were then mounted on a microscopic slide and covered with a 15mm coverslip for confocal imaging using Zeiss confocal LSM800 (Zeiss LSM800, Germany). The selected fluorescent dyes—Calcofluor White and DAPI (4’,6-diamidino-2-phenylindole)—were utilized to investigate cell walls and nuclei, respectively. For each sample, 3–5 fields per slide were imaged from 3 biological replicates. The mean fluorescence intensity (MFI) for each field was obtained after subtracting the background fluorescence. Nuclear size was quantified at 350/470 nm excitation/emission to detect DAPI. Cell wall integrity and structure were determined at 410/455 nm excitation/emission in Calcofluor White-labeled samples. Images were analyzed using Zeiss Zen Blue 2.3 image processing software (Zeiss, 2016).

### Elemental analysis

The pH and electrical conductivity (EC) of the liquid growth media were assessed using Vernier sensors (Vernier, OR, USA) to elucidate the impact of PFOA on the chemical properties of water. To investigate potential interactions between PFOA and cations, the concentrations of selected elements were determined in plants exposed to 0 and 0.1 µg/L PFOA for seven days using an inductively coupled plasma mass spectrometer (ICP-MS) (350X NEXION, Perkin Elmer, USA). For sample preparation, acid digestion was carried out following the EPA 3050B Method (U.S. EPA, 1996) with modification for plant samples as described in Noori et al. (2020). *Lemna minor* samples were transferred into 50 mL polypropylene tubes and were dried in an oven at 50°C for 48 hours or until completely desiccated. After recording the dry weight of each sample, 5 mL of HNO_3_ was added to each tube. The tubes were covered with polypropylene watch glasses and placed on the DigiPREP Block (SCP Science DigiPREP, Canada) at 98°C for 30 minutes. Subsequently, 1 mL of 30% H_2_O_2_ was added to each tube, and samples were heated for an additional 20 minutes or until complete digestion. The contents of each tube were filtered using Whatman #1 filter paper, and the samples were diluted with ultrapure water to a final volume of 50 mL. Calibration standards at concentrations of 0, 0.1, 1, 10, 50, and 100 µg/L were prepared using a multi-element calibration solution (Perkin Elmer, Catalog # N9301721), with 0 and 10 µg/L calibration solutions used for quality control. To assess the impact of filtration on the samples, an additional blank solution was periodically filtered and analyzed. All samples, including calibration standards, were spiked with 5 µg/L of a multi-element internal standard containing Bi, In, Sc, and Y (Perkin Elmer, Catalog #N9303834) to enhance precision and accuracy. The cation content in each tube was determined by ICP-MS 350X NEXION (Perkin Elmer, USA) and reported as µg/L for the growth media and µg/g dry weight for plants. The impact of PFOA exposure on the concentration of detected elements in plants was analyzed using the Pearson correlation coefficient at a 95% confidence level.

### PFOA extraction and analysis

To detect PFOA in the plant tissues and growth media, PFOA (>98% purity) and perfluoro-n- (^13^C_8_) octanoic acid (M8PFOA) (>98%) were purchased from Sigma Aldrich and Wellington laboratories, respectively. The stock solution of PFOA was prepared in HPLC-grade methanol (Fisher Chemicals). Ultrapure water was obtained from an in-house Milli-Q Integral 5 water purification system. Ammonium acetate and Supelclean ENVI-Carb 120/400 were purchased from Sigma Aldrich.

Plant tissue PFOA was extracted based on our previous work (Nason et al., 2024). The plant samples were oven-dried, and then 0.1 g of the dried samples were homogenized with a ceramic mortar and pestle. All samples were spiked with the ^13^C8-PFOA at 1 ng/mL in the final extract and were equilibrated overnight prior to extraction. Samples were extracted three times with 4 mL of methanol containing 400 mM ammonium acetate. Each extraction consisted of 5 minutes of vigorous shaking on a paint can shaker followed by 5 minutes centrifugation at 3000 rpm. Supernatant from the three extractions were combined and evaporated under N_2_ in a 60°C water bath, then reconstituted to 2 mL with methanol and vortexed. Extracts were transferred to polypropylene tubes containing 40 ± 5 mg of ENVI-Carb and vortexed followed by centrifugation at 14,000 rpm for 30 minutes. A 750 µL aliquot of the supernatant was filtered through a 0.2 µm regenerated cellulose membrane, centrifuged at 10,000 rpm for 10 minutes, then transferred to a polypropylene autosampler vial. A spiked control sample and a method blank were extracted alongside each batch of samples. The growth media samples were centrifuged at 14000 rpm for 10 minutes, filtered through a 0.2 µm regenerated cellulose membrane and transferred to a polypropylene autosampler vial.

PFOA was measured using liquid chromatography coupled with triple quadrupole mass spectrometry (LC-MS/MS) (Agilent, CA, USA). Chromatography was performed using an Agilent 1690 ultra-high-performance LC equipped with a PFAS delay column and a Thermo Hypersil Gold C-18 column (100 mm x 2.1 mm, 1.9 µm particles) with an Accucore aQ guard column (10 mm x 2.1 mm, 2.6 μm particles) (**Supplementary Table S2**). Mobile phases were 0.1% formic acid in ultra-pure water (A) and 0.1% formic acid in acetonitrile (B). The injection volume was 2 µL and the flow rate was 300 µL/min. The column oven was kept at 40°C and the autosampler at 10°C. The solvent gradient went from 20% B to 100% B, and each run was 23 minutes (**Supplementary Table S3**). Quantification was performed using a SciEx 7500 triple quadrupole mass spectrometer. Negative electrospray ionization was used. The calibration range was 0.01 to 100 ng/mL. All standards contained the same ^13^C8-PFOA concentrations as the samples for each run (**Supplementary Table S4**). Every 10 to 15 samples, a solvent blank and a standard solution were analyzed to track instrument performance. Quantitative analysis was performed using SciEx OS software. Calibration curves weighed 1/x. Automated MQ4 peak integration was used, and integrations were manually curated to ensure accuracy. Bioconcentration factors (BCF) for PFOA in *L. minor* were calculated using the following equation:


$$BCF=\frac{Conc._{Plant}}{Conc._{Growth\;Media}}$$

where *Conc. _Plant_* is the PFOA concentration in plant dry weight (ng/g) and *Conc. _Growth Media_* is the PFOA concentration in the aquatic growth media.

### Statistical analysis

A minimum of three replicates per group were used for all experiments. To assess the normality of the data, Shapiro-Wilk tests were conducted. Subsequently, based on the results of the normality tests, either a T-test or one-way ANOVA followed by Tukey’s test with a confidence level of ρ ≤ 0.05 was applied. In cases where a statistical difference among the treatment means was observed, LSD and Tukey tests at a 5% significance level were employed for equal variances using SPSS 29 (IBM Corp, 2024, NY). The graphs were generated using GraphPad Prism version 10.0 for Windows (GraphPad Software, Boston, MA) (GraphPad Software, Inc, 2024).

## Results and discussion

### Half-maximal inhibitory concentration (IC_50_) and time-dependent phytotoxicity

The IC_50_ assay results revealed a correlation between PFOA concentration and more pronounced phytotoxic responses of *L. minor*, leading to an up to 80% reduction in plant growth upon exposure to 100 µg/L PFOA. The 0.1 µg/L treatment level was identified as the threshold leading to less than a 50% reduction in plant growth (75 ± 2.8%) (**Figure 1A**) and chlorosis (52.63 ± 4.2%) compared to controls.

To evaluate the phytotoxic effects of PFOA exposure over time, the fronds of *L. minor* exposed to 0 (C) and 0.1 µg/L PFOA for 1, 7, and 14 days were evaluated (**Figure 1B**). The chlorosis index increased for both groups from day 1 to day 14, with a significant difference (ρ=0.02) observed between the control (C) and PFOA (P) groups on day 14 of exposure with the values of 15.29 ± 2.85% and 26.35 ± 5.09%, respectively. This indicates a 25% increase in chlorosis compared to day one and a 15% higher level than the control group on day 14. These findings suggest time- and dose-dependent responses of *L. minor* to PFOA. Dose-dependent phytotoxicity responses are expected and have been commonly reported by others. Chen et al. (2020) exposed *Arabidopsis thaliana* and *Nicotiana benthamiana* to 5 and 20 mg/L of PFOA in MS medium agar for 21 days. They reported concentration-dependent responses based on tolerance index (TI) values that were determined using root length, root weight, and shoot weight. This study showed significant inhibition in the development of roots and shoots upon exposure to 20mg/L PFOA while exposure to 5 mg/L only inhibited the growth of their shoots. Similarly, Qian et al. (2019) studied the phytotoxicity of *Acorus calamus* and *Phragmites communis* upon exposure to 1 - 50 mg/L PFOS, a long chain and common PFAS, in a hydroponic media for 48 days. Exposure to 10 and 50 mg/L PFOS for 48 days prevented chlorophyll concentration by 13.7 – 22.2% and 22.4-30.0%, while exposure to lower concentration had no negative impact on chlorophyll content. They also reported more changes in biomarkers such as photosynthetic pigments, proteins, oxidative stress indicators, and antioxidative responses during days 0-24 of the experiment compared with days 24-48. They suggested time and concentration-dependent phytotoxicity responses.

### Physiological responses to PFOA exposure

**Figure 2:**
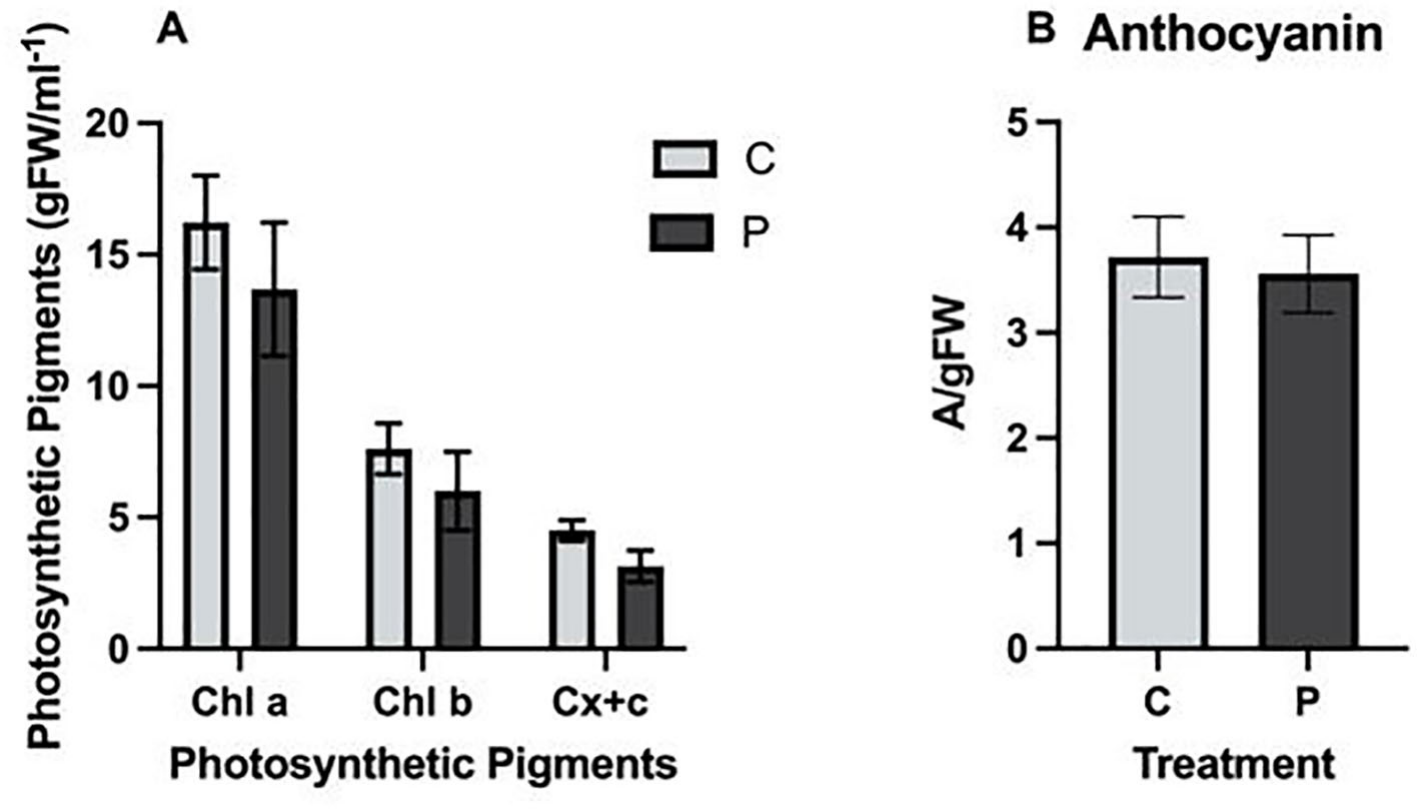
**(A)** Photosynthetic pigments Chla, Chlb, and carotenoids (Cx+c), and **(B)** Anthocyanin Content in *L. minor* exposed to 0.1µg/L PFOA (P) for seven days compared to the control group (C). The bars represent the mean ± SE of three biological replicates. Statistical significance was determined using a T-Test with a confidence level of ρ ≤ 0.05.

The concentration of Chla, Chlb, and carotenoids was unaffected by exposure to PFOA. Specifically, the amount of photosynthetic pigments in *L. minor* grown in 0 or 0.1 µg/L PFOA were Chla: 16.22 ± 1.78, 13.68 ± 2.54; Chlb: 7.60 ± 0.97, 6 ± 1.49; and carotenoids: 4.50 ± 0.39, 3.14 ± 0.6/gFWml^-1^, respectively (**Figure 2A**). Similarly, the anthocyanin content of *L. minor* was unaffected by PFOA exposure. The control and 0.1 µg/L PFOA exposed groups had values of 3.72 ± 0.22 A/gFW and 3.56 ± 0.21 A/gFW, respectively (ρ=0.632) (**Figure 2B**). The oxidative stress indicator analysis showed that exposure to PFOA did not significantly impact hydrogen peroxide or MDA content. Hydrogen peroxide levels in the control and experimental groups were 0.064 ± 0.0002 and 0.064 ± 0.003 M/g FW, respectively (ρ=0.673) (**Supplementary Figure S1A**). The MDA content of the control group was 0.57*10^3^ ± 0.03 mM, while the PFOA-exposed group was 0.53*10^3^ ± 0.01 mM (**Supplementary Figure S1B**) (ρ=0.27).

Examining photosynthetic pigment content offers valuable insights into early signs of stress, toxicity, and overall plant health. The negligible differences observed in the photosynthetic pigment content between the control and experimental groups in our study suggest that exposure to 0.1 µg/L does not result in a significant negative impact on *L. minor*. This finding aligns with the study of Pietrini et al. (2019), who also reported no significant impact on the photosynthetic content of *L. minor* exposed to 2 µg/L of PFOA over seven days. In contrast, Lan et al. (2018) observed a 124% increase in Chla content in wheat exposed to 200 µg/kg PFOA in soil for four weeks under greenhouse conditions. Meanwhile, exposure to C4 compounds resulted in a 25% decrease in chlorophyll levels, highlighting the differential impact of various types of PFAS. The differences in species, growth medium, and experimental conditions are likely contributing factors to the variation in results. Unlike wheat, *Lemna minor* grows in an aquatic environment, which may influence PFOA uptake and distribution. Additionally, *Lemna minor* is known for its high tolerance to environmental pollutants due to its rapid growth, efficient antioxidant mechanisms, and ability to adapt to stress, which could mitigate the impact of PFOA on photosynthetic pigments. Adu et al. (2023) noted that the effect of PFAS compounds on photosynthetic pigments depends on the specific compound and plant species. In the current study, exposure to PFOA did not significantly impact oxidative stress indicators and antioxidative stress responses. A similar observation was made by Zhang and Liang (2021), who investigated the effects of 10 µg/L PFOS on *L. minor* over 7 and 14 days and found no significant differences in the activity of antioxidative enzymes, specifically superoxide dismutase (SOD) and peroxidase (POD), between the experimental and control groups. Separately, Li et al. (2021) examined lettuce (*Lactuca sativa*) under hydroponic exposure to 5 µg/L PFOA and reported no impact on oxidative stress responses. However, exposure to 50 µg/L resulted in a significant induction of oxidative stress indicators (H_2_O_2_ and MDA) and antioxidative enzymes (e.g., CAT, SOD, POX), demonstrating clear dose-dependent phytotoxicity. Gonzales et al. (2023) also reported that *L. minor* exposed to 0.3 and 3 µg/L PFOA for seven days exhibited higher oxidative stress responses at 3 ppb PFOA. Mechanistically, elevated levels of reactive oxygen species (ROS) such as H_2_O_2_ damage macromolecules such as lipids, proteins, and nucleic acids, potentially leading to organelle damage and even cellular death. Wu et al. (2023) reported alterations in DNA, lipids, and proteins in *L. minor* exposed to PFOA at concentrations ranging from 1 to 100 µg/L for 96 hours. Their findings also emphasize the dose- and type-specific impacts of PFAS, particularly at higher concentrations of PFOA and PFOS. Importantly, the negligible phytotoxic responses of *L. minor* detected in this study at environmentally relevant doses suggest its potential feasibility for use in phytoremediation.

### Stomatal pore size

**Figure 3:**
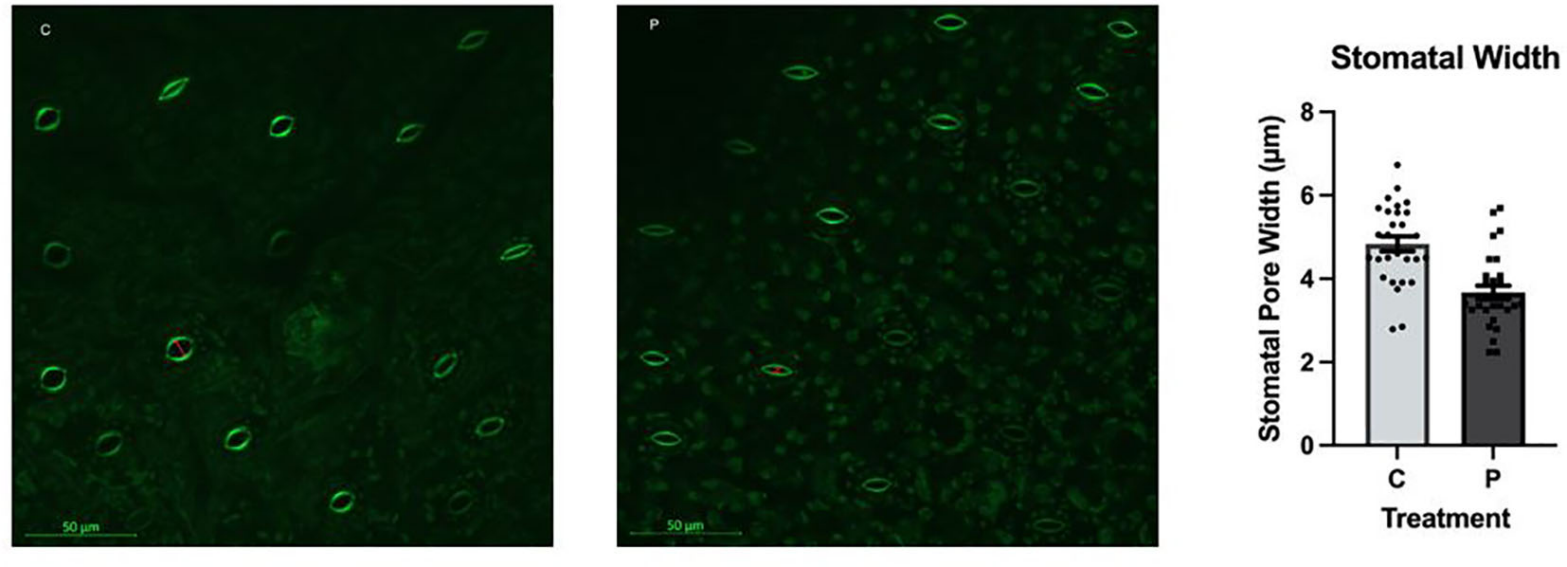
Stomatal pore structure and width in *L. minor* in the control group (C) or exposed to 0.1 µg/L PFOA (P) for 7 days. The bars depict the mean ± SE of 30 replicates. The data passed the Shapiro-Wilk normality test. Statistical significance was assessed using a T-Test with a confidence level of ρ ≤ 0.05 (ρ<0.0001).

The average stomatal pore size was significantly different between the control and the PFOA-exposed groups (ρ < 0.0001); the values were 4.839 ± 0.24 µm and 3.672 ± 0.68 µm, respectively (**Figure 3**). Stomata play a critical role in gas exchange, water transpiration, photosynthesis, and overall plant health. The wider opening of stomatal pores in the control group compared with the PFOA-exposed group suggests a lower transpiration rate, gas exchange, and potentially reduced photosynthesis rate upon contaminant exposure. This aligns with the growth and physiological data, indicating an association between the higher rates of chlorosis, lower growth rate, and the narrower stomatal pores occurring with contaminant exposure. While exposure to 0.1 µg/L PFOA did not significantly impact the photosynthetic pigment content, the observed 3% reduction after seven days suggests a moderate level of toxicity in *Lemna minor*. This indicates that some physiological parameters are more sensitive to PFOA exposure than others. Prolonged exposure or higher concentrations of PFOA may lead to a greater loss of photosynthetic pigments and more pronounced chlorosis.

Battisti et al. (2023) measured the stomatal conductance of willow using a Li-600 Li-Cor device, and reported a reduction in the opening of stomatal pores upon exposure to 100 µg/L of a mixture of different types of PFAS for 8 days. PFOA can impact plant stomata similarly to other organic or inorganic environmental stressors. Specifically, exposure to abiotic stresses can induce stomatal closure, leading to decreased stomatal conductance and reduced evapotranspiration (Miras-Moreno et al., 2022; Li S. et al., 2022). The smaller stomatal openings observed in PFOA-exposed *L. minor* may be related to the impact of PFOA on the plant’s growth media, affecting the availability and uptake of essential elements like potassium (K), which play a critical role in regulating the opening and closure of stomatal pores. The elemental analysis data which is discussed further below revealed that concentration of K in *L. minor* was reduced by nearly 40% upon exposure to PFOA. This supports the narrower size of stomatal pore in the experimental group.

### Cell size and cell wall structure

**Figure 4:**
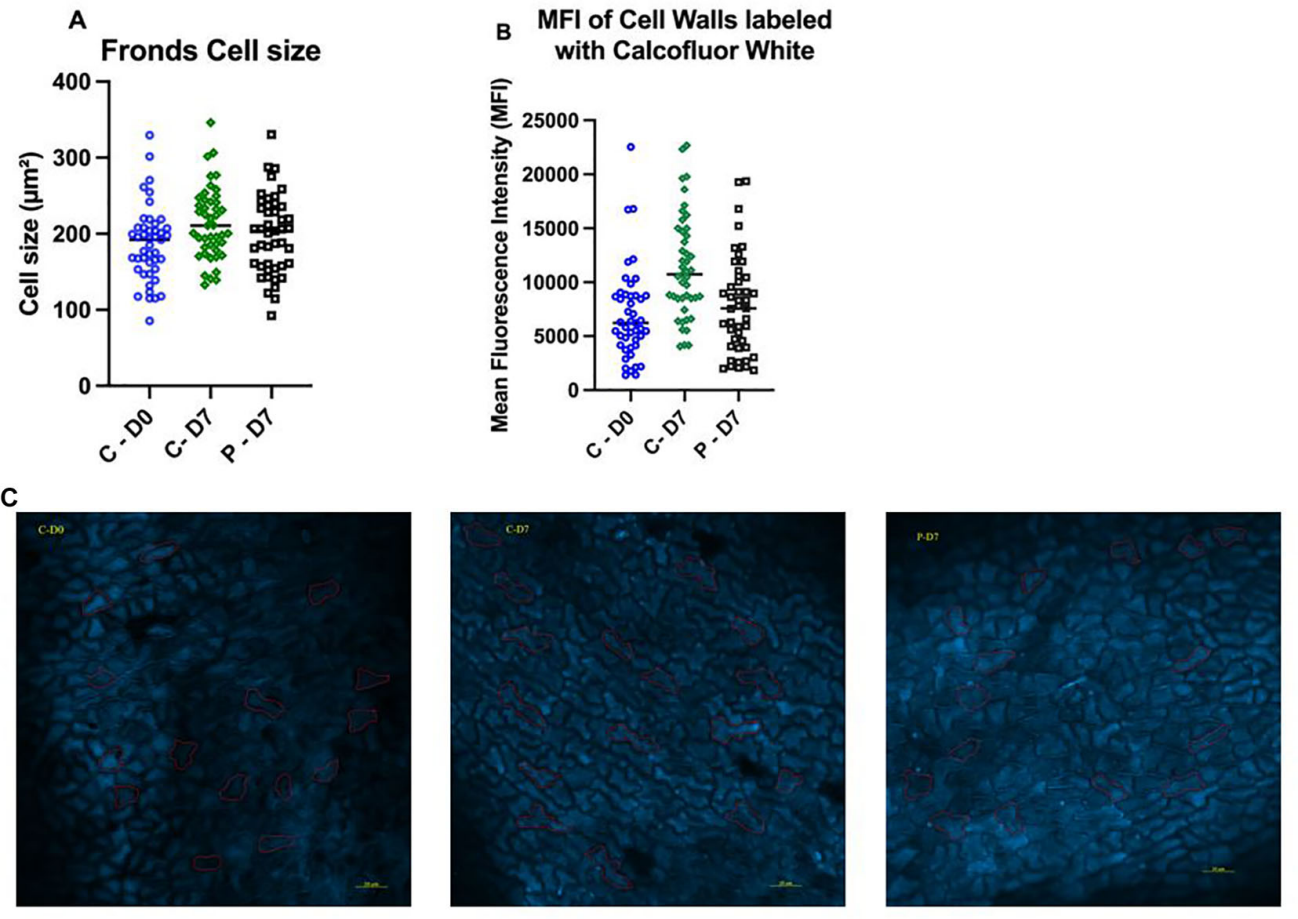
**(A)** Cell size of *L. minor* fronds prior to exposure (C-D0), after seven days of growth in chlorine-free water (C-D7), or after exposure to 0.1 µg/L PFOA (P-D7). **(B)** Mean fluorescence intensity (MFI) of cell walls stained with calcofluor white. **(C)** Microscopic images of cell walls labeled with calcofluor white and imaged at 200x magnification using a Zeiss LS800 confocal microscope with 410/455 nm Ex/Em. Quantification of MFI and representative images were obtained from 3 to 5 fields per slide from 3 biological replicates. Bars represent the mean ± SE of 45 cells recorded from each group. The data passed the Shapiro-Wilk normality test and were analyzed using One-Way ANOVA followed by Tukey’s test with a confidence level of ρ ≤ 0.05.

*Lemna minor* labeled with calcofluor white underwent immunofluorescent imaging to record both average cell size and cell wall structure in the fronds. The average cell size area in the controls (213 ± 6.5 µm²) after seven days (C-7) was significantly higher than cells at day zero (D0) (186 ± 18 µm²) (ρ = 0. 02); however, no significant differences were recorded between the group exposed to 0.1 µg/L PFOA (198 ± 13 µm²) for seven days (P-7) and the D0 group (ρ = 0. 4) (**Figure 4A**). The mean fluorescence intensity (MFI) data of epidermal cells labeled with calcofluor white showed that the controls (C-7) had significantly higher values (11429 ± 1726 MFI) compared with those exposed to 0.1 µg/L PFOA (7864 ± 443 MFI) (ρ = 0.0008), as well as compared to the day zero (6978 ± 1062 MFI) plants (ρ < 0.0001) (**Figures 4B, C**).

High MFI values in calcofluor-labeled areas indicate greater concentrations of cellulose, which correspond to actively growing regions. This aligns with the observed increase in average cell size in the control group after seven days. The significantly higher MFI and cell size in the control group suggest robust growth and cell wall development, while the lower values in the PFOA-exposed group suggest inhibitory effects of PFOA exposure. The day zero measurements serve as a baseline, showing that the control group has a marked increase in both cell size and cellulose content over time, whereas the PFOA group shows more limited growth and development.

### Nucleus diameter and size

**Figure 5:**
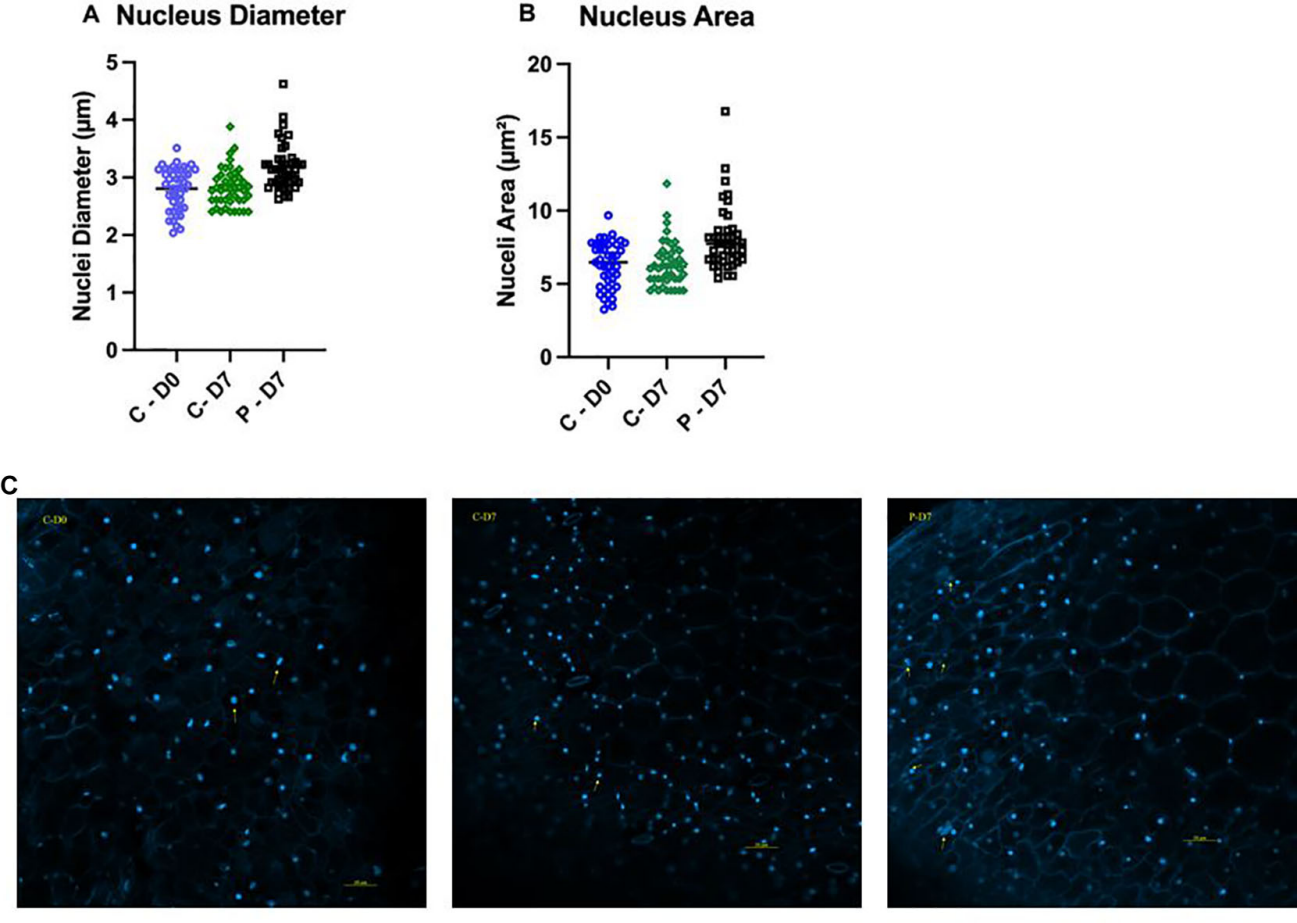
*Lemna minor* epidermal cell nuclear diameter and size prior to exposure (D0), after seven days of growth in chlorine-free water (C-7), or after exposure to 0.1 µg/L PFOA (P-7). **(A)** Comparison of nuclear diameter between the three groups. **(B)** Comparison of nuclear area size between D0, C-7, and P-7. **(C)** Nuclei labeled with DAPI and imaged at 200x magnification using a Zeiss LS800 confocal microscope with Ex/Em at 350/470 nm. Data were collected from three fields per slide from three biological replicates. Bars represent the mean ± SE of 45 cells recorded from each group. The data passed the Shapiro-Wilk normality test and were analyzed using One-Way ANOVA followed by Tukey’s test with a confidence level of ρ ≤ 0.05.

Both the nuclear diameter and area increased significantly after seven days of exposure to PFOA compared with the controls and the day zero (D0) samples (ρ < 0.0001). The average nuclear diameter on the initial day of the experiment (D0), as well as in the controls (C-7) and in the PFOA-exposed group after seven days (P-7) were 2.8 ± 0.14 µm, 2.85 ± 0.1 µm, and 3.17 ± 0.13 µm, respectively (**Figure 5A**). This indicates that exposure to 0.1 µg/L PFOA for seven days significantly increased the nuclear diameter by 13% compared to both D0 and C-7. Similarly, the nuclear area significantly increased upon exposure to 0.1 μg/L PFOA (8.02 ± 0.7 µm²) compared to the control group at day seven (6.33 ± 0.45 µm²) and day zero plants (6.28 ± 0.64 µm²) (ρ < 0.0001) (**Figure 5B**). The increase in nuclear size suggests alterations in nuclear differentiation and cellular development. Interestingly, a higher number of cells were at the anaphase stage in the group exposed to PFOA compared to the controls (**Figure 5C**), indicating an impact on cellular division. Although research on the impact of PFOA on plant nuclei and cell division is limited, similar effects have been reported in mammalian cells exposed to various types of PFAS, including PFOA. Wen et al. (2020) reported alterations in the cell cycle and DNA methylation in mice exposed to 1-20 mg/kg PFOA per day for 10 days, with dose-dependent effects resulting in more profound changes in nucleic acid expression and cell cycle regulation. Zhao et al. (2022) observed a significant increase in the size of the nucleus in human neuroblastoma SH-SY5Y cells exposed to 0.4 and 4 μg/L PFOA for 96 hours, with nuclear size increasing by 6% and 11%, respectively, compared to the control group. The authors proposed that proliferation of PFOA exposed cells and the increased rate of transition from G1/G0 to S has contributed in the nuclear size as cells in S phase show larger nuclear size compared to cells in interphase. Although these reports focus on mammalian cells, the similarity of eukaryotic organisms at the cellular level allows us to interpret our results using these findings. The results of our study clearly suggest that PFOA exposure disrupts normal cellular processes in *Lemna minor*, leading to increased nuclear size and altered cell division, potentially mirroring similar impacts on mammalian cells. However, further investigation is needed to better understand the mechanisms underlying PFOA impact on plant cells at the nuclear level.

### Elemental analysis

**Figure 6:**
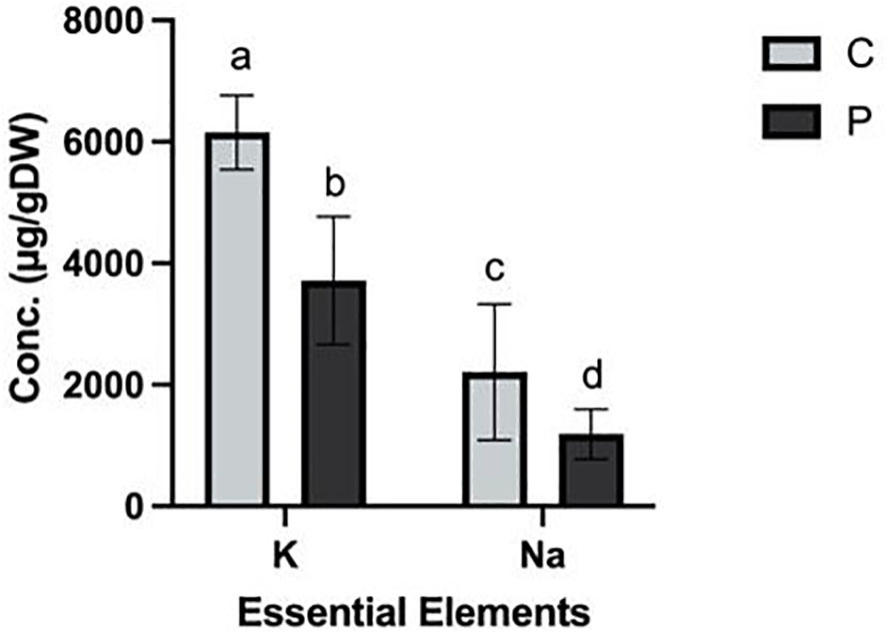
Elemental analysis of K and Na in *L. minor* exposed to 0 (C) and 0.1µg/L (P) PFOA for seven days. The bars depict the mean ± SE of three biological replicates. Different letters denote significant differences, and statistical significance was determined using One Way ANOVA followed by Tukey’s test with a confidence level of ρ ≤ 0.05.

Elemental analysis of the plant tissues revealed significant differences in the concentrations of K (ρ = 0.000) and Na (ρ = 0.006) between the control (C) and experimental (P) groups. Na levels were 2209 ± 647 μg/g and 1186 ± 237 μg/g in the control and exposed plants, respectively. Similarly, the K concentrations were 6154 ± 351 μg/g and 3717 ± 609 μg/g in control and exposed groups, respectively (**Figure 6**). No statistically significant differences were observed in the concentrations of other measured elements (**Supplementary Figures S2A, B**). The reduced concentration of K detected in the PFOA-exposed group can explain the narrower stomatal openings in this group. Specifically, optimum concentration of K^+^ in guard cells is crucial for maintaining turgor pressure and facilitating the opening of stomatal pores (Cochrane and Cochrane, 2009; Misra et al., 2019). The lower concentration of sodium may influence the balance of other elements, turgor pressure, or H^+^-ATPase activity, which could disrupt ion transport and overall cellular homeostasis.

A Pearson correlation analysis of several elements in *L. minor* exposed to PFOA showed significant relationships. Specifically, there was a significant negative correlation (ρ ≤ 0.05) between Na and K, Ca, Fe, and Mo in the PFOA-exposed group. Additionally, K exhibits a significant positive correlation with Ca, Fe, and Mo (**Supplementary Table S5A**). These correlations were not observed in the controls (**Supplementary Table S5B**). After seven days, the pH of controls remained neutral, exposure to PFOA resulted in the reduction of pH from 7.8 ± 0.22 to 7.3 ± 0.044. The significantly reduced concentrations of K and Na observed in *L. minor* exposed to PFOA may be attributed to this slight decline in pH. The lower pH recorded in the growth media of the experimental group is likely a consequence of the release of H^+^ by PFOA. This acidic environment could lead to a heightened positive electrical charge in the growth media, potentially influencing the activity of the transporter H^+^-ATPase and subsequently affecting the uptake and transportation of elements across the membrane. However, the expression or functionality of H^+^-ATPase or other potential mechanisms involved in the absorption and transportation of cations in *L. minor* were not investigated in this study but are a topic of ongoing investigation. While concentrations of Ca, Cu, Fe, Mo, and Zn, were not significantly impacted, the trend of decreased content could become significant over a longer exposure period. Additional research is necessary to investigate the long-term effects and implications of PFOA exposure on the overall elemental uptake and nutritional homeostasis in *L. minor*.

### PFOA analysis

**Figure 7:**
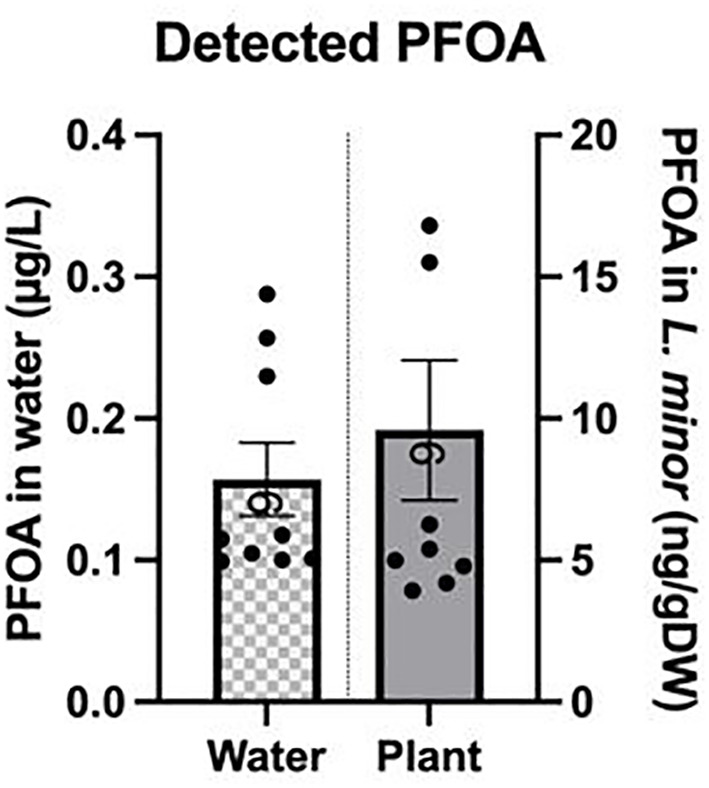
PFOA concentration detected in the growth media and *L. minor* exposed to 0.1μg/L PFOA for seven days. The bars depict the mean ± SE of nine biological replicates.

PFOA was detected in *L. minor* exposed to 0.1 µg/L for seven days; the average bioaccumulation factor was 56 ± 7. The detected concentration of PFOA in the growth media and *L. minor* upon exposure to 0.1 µg/L of PFOA was 0.16 ± 0.025 µg/L and 9.58 ± 2.4 ng/gDW, respectively (**Figure 7**). The higher than exposed concentration of PFOA detected in the growth media may be attributed to factors such as evaporation or the potential presence of PFOA in laboratory materials, leading to the observed increase. Additionally, it is important to note that even though no PFOA was used in the control group and only chlorine-free water was used to grow *L. minor*, a small level of PFOA was detected in some of the samples, resulting in an average concentration of 0.0154 ± 0.005 µg/L and 0.65 ± 0.052 ng/gDW in the growth media and *L. minor*, respectively. This resulted in a bioaccumulation factor of 0.02 ± 0.01 in the controls. To identify the source of PFOA in the control group, water used for plant growth was analyzed and found to be below the detection limit (LOD). Despite careful experimental design and analysis, the detected PFOA levels in the control group could be attributed to contamination on the surfaces of materials used during the experiment or cross-contamination through the volatilization of PFOA from the experimental group. Additionally, ambient environmental contamination or cross-contamination during handling could have contributed to this small level of PFOA detected in the control samples.

The potential of plants to remove PFAS has been shown to vary with the growth environment, plant species, and the specific PFAS compound. For example, Zhang and Liang (2021) found that the ability of *L. minor* to remove PFAS from the growth medium depended on the properties of the specific contaminant. Specifically, *L. minor* exposed to 200 µg/L of perfluorooctanesulfonamide (PFOS) and 6:2 fluorotelomer sulfonate (FTS) for seven days showed significantly higher uptake of FTS (285.54 ± 86.13 ng) compared to PFOS (14.81 ± 1.43 ng). This suggests that the higher water solubility of FTS contributed to its greater accumulation in plants. Our findings are comparable, considering the similarities between PFOA and PFOS.

Greger et al. (2021) studied the potential of several species, including the emergent plants *Carex rostrata* and *Euriophorum angustifolium*, and *Elodea canadensis as a* submerged plant, for PFOA removal from an aquatic environment. The authors observed higher bioaccumulation of PFOA in emergent plants compared to the submergent species *E. canadensis*. The bioaccumulation of PFOA by *C. rostrata* during the 12-day treatment reached up to 42% of the total amount plants were exposed to. Additionally, an increase in the bioaccumulation of PFOA by both *E. canadensis* and *C. rostrata* over time was noted. Our findings align with this work *as L. minor*, an emergent species, showed the bioaccumulation factor of 56 upon exposure to 0.1 µg/L PFOA in water. Greger and Landberg (2024) recently reported on the phytoremediation of emerging compounds from contaminated water collected from Sänksjön Lake in Sweden using several aquatic species. The authors showed that plants with higher biomass were able to bioaccumulate more PFOA; for example, 7.91% uptake of PFOA from water by *Carex elata* was reported after only 96 hours of cultivation in PFAS-contaminated water. When considering phytoremediation for the removal of PFAS, it is important to note that the bioaccumulation of PFAS by aquatic species differs from that by terrestrial plants. Most studies on the potential of terrestrial species to remove PFAS from soil report lower levels of bioaccumulation over a longer timeframe. This could be related to the fact that the ability of terrestrial plants to remove PFAS from soil is dramatically influenced by the high complexity of the soil environment, including soil physicochemical properties and organic components of the soil, as well as plant species differences (Melo et al., 2022). Nassazzi et al. (2023) reported on the potential of sunflower (*Helianthus annuus)*, mustard *(Brassica juncea)*, and hemp (*Cannabis sativa*) grown in soil in bioaccumulating PFAS and noted that the efficacy of PFAS phytoremediation depends on plant species and type of the PFAS. Specifically, the bioaccumulation factor of PFOA by hemp ranged from 6 to 41, while mustard and sunflower bioaccumulation ranged from 4.8-14 and 3.3-15, respectively. He et al. (2023) studied the efficacy of seven herbaceous species belonging to *Compositae* or *Poaceae* family to remove various types of PFAS from soil. The authors found that the studied plants accumulated 0.8 - 8% PFOA in their shoots, a significantly lower amount compared to light-chain PFAS, which exhibited an approximate removal rate of 44% from the soil. The authors suggested the chemical structure of PFOA as a long chain PFAS reduces its bioaccumulation compared with shorter chain molecules like Perfluoro-3-methoxypropanoic acid (PFMOPrA). Future work should consider additives beyond surfactants, including engineered nanomaterials, to enhance phytoaccumulation of PFAS that aren’t naturally accumulated (Lewis et al., 2023). In addition, while this study focuses on the short-term effects of PFOA exposure on *Lemna minor*, it is important to consider the potential long-term implications for this species and similar aquatic plants. Prolonged exposure to PFOA could exacerbate oxidative stress, leading to cumulative damage to cellular structures, including membranes and chloroplasts, which may result in chronic reductions in photosynthetic efficiency and growth. Over time, this could decrease the plant’s ability to sustain its health, potentially impacting the entire aquatic ecosystem where *L. minor* or other affected aquatic species serves as a primary producer. Future research focusing on long-term studies is essential to better understand these potential impacts and to provide a more comprehensive assessment of how PFOA affects not only *L. minor* but also the ecosystems it supports.

## Conclusion

The physiological responses of *L. minor* to PFOA exposure and the potential of this species to remove this chemical from a model aquatic environment were investigated. The findings of this study highlight the applicability of *L. minor* for phytoremediation strategies, its resilience to pollutants, and ability to accumulate contaminants like PFOA in its tissues. The species’ rapid growth and adaptability further support its use as a cost-effective and environmentally friendly option for remediation of PFOA-contaminated water bodies. The results show that *L. minor* can tolerate exposure to 0.1 µg/L for seven days with negligible phytotoxicity and is able to bioaccumulate more than 50% of PFOA available in the growth media. Although exposure to 0.1 µg/L PFOA resulted in a 50% growth reduction compared with the control group, achieving 50% of contaminant-free growth is a reasonable compromise that still allows for significant phytoremediation. These results are promising for the phytoremediation of PFOA from aquatic systems, particularly from surface water where PFOA is commonly detected. Although the ability of plant species to bioaccumulate PFAS in their tissues is a concern from an environmental and human health perspective, if nonedible plants are chosen and safety guidelines are followed, the current work suggests that phytoremediation may be a viable strategy for the removal of PFAS from aquatic environments. The properties of *L. minor* as a rapidly growing species with potential to tolerate environmental pollutants provide additional value in phytoremediation practices.

This study offers a preliminary understanding of the impact of PFOA, a selected long-chain PFAS, on *Lemna minor* as an aquatic species. However, further studies are needed to better understand the bioaccumulation mechanism of PFOA and other PFAS analytes, as well as the molecular-level impacts of exposure on plant species over longer periods of time at various doses.

## Data Availability

The original contributions presented in the study are included in the article/**Supplementary Material**. Further inquiries can be directed to the corresponding author.
