# Supplementary material for: Phytotoxicity and phytoremediation potential of Lemna minor exposed to perfluorooctanoic acid
Source: Front Plant Sci. 2025 Jan 27;15:1493896. doi: 10.3389/fpls.2024.1493896 (PMC11807973; doi:10.3389/fpls.2024.1493896)
Supplement: Supplementary file 1 [file SupplementaryFile1.docx]

**Supplementary Information**

**
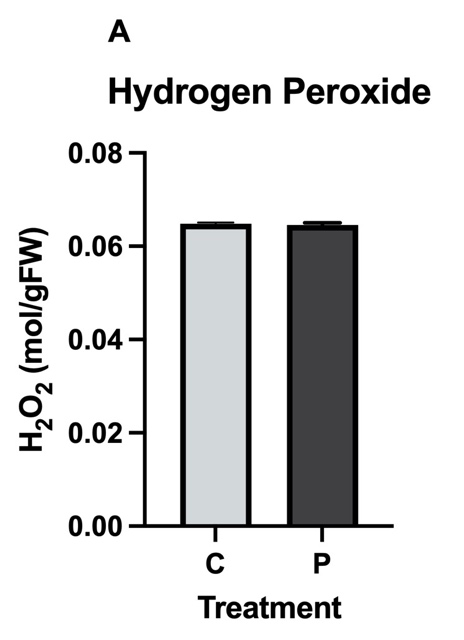

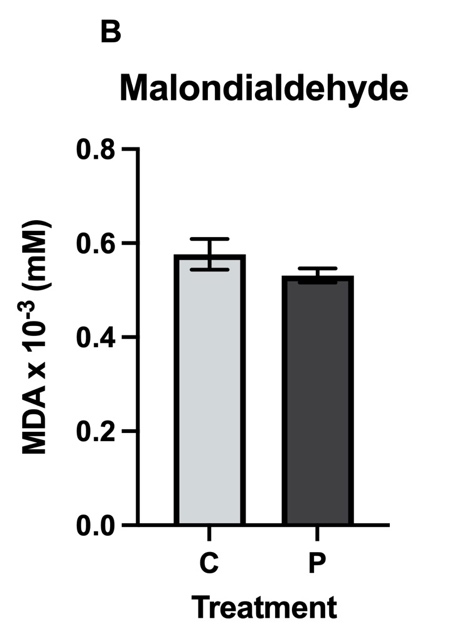
**

Figure S1. Oxidative stress indicators. A) Hydrogen Peroxide, B) Lipid Peroxidation in *L. minor* cultivated in chlorine-free water (C) or exposed to 0.1 µg/L PFOA (P) for seven days. The bars depict the mean ± SE of three biological replicates. Statistical significance was determined using a T-Test with a confidence level of ρ ≤ 0.05.

| **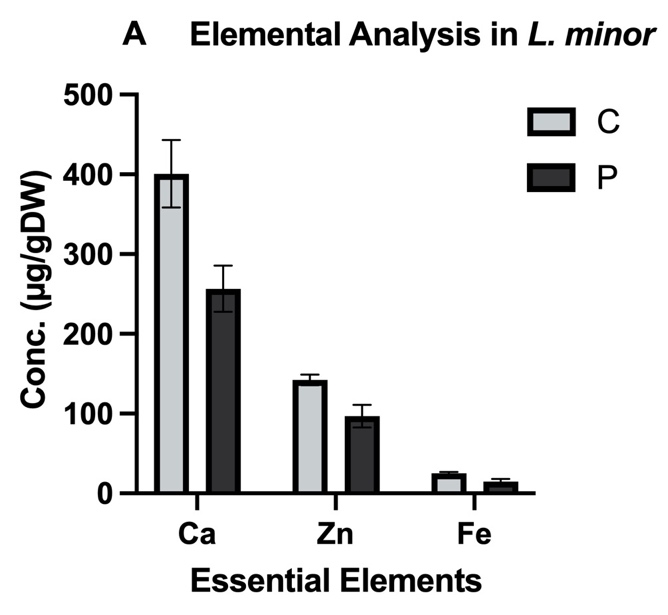** | **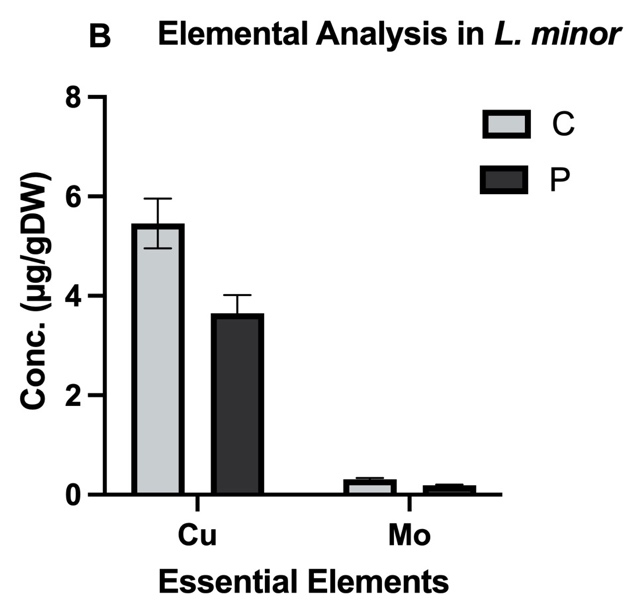** |
| --- | --- |

Figure S2. Detected concentration of elements A) Ca, Zn, Fe and B) Cu and MO in the (C) control and exposed *L. minor* to (P) 0.1µg/L PFOA for seven days. The bars depict the mean ± SE of three biological replicates. Statistical significance was determined using a T-Test with a confidence level of ρ ≤ 0.05.

Table S1-The fluorescent dyes working concentration for live cell imaging

| **Fluorescent Dye** | **Final Conc. (µg/mL)** | **Treatment pH** | **Excitation/Emission (nm)** |
| --- | --- | --- | --- |
| Calcofluor white | 1 | 7 | 410/455 |
| DAPI | 10 | 7 | 350/470 |

Table S2-PFOA detection settings

| **Compound** | **Parent ion** | **Quantitative Ion** | | | **Qualitative Ion** | | | | **RT (min)** |
| --- | --- | --- | --- | --- | --- | --- | --- | --- | --- |
|  |  | **Mass** | **CE (V)** | **CXP (V)** | **Mass** | **CE (V)** | | **CXP (V)** |  |
| PFOA | 413 | 369 | -15 | -19 | 169 | -23 | -12 | | 10.23 |
| 13C8-PFOA | 421 | 376 | -15 | -17 | 172 | -22 | -12 | | 10.24 |

Table S3-LC Solvent Gradient

| **Time (min)** | **%B** |
| --- | --- |
| 0 | 20 |
| 2.5 | 20 |
| 2.51 | 30 |
| 6 | 30 |
| 6.01 | 40 |
| 9 | 40 |
| 9.01 | 50 |
| 12 | 50 |
| 12.01 | 65 |
| 14.5 | 65 |
| 14.51 | 85 |
| 17 | 85 |
| 17.01 | 20 |
| 18.5 | 100 |
| 18.51 | 20 |
| 21 | 20 |

Table S4-SciEx 7500 settings

| Polarity | Negative |
| --- | --- |
| Ion source gas 1 | 40 psi |
| ion source gas 2 | 50 psi |
| Curtain gas | 40 psi |
| CAD gas | 12 |
| Source temperature | 550 C |
| Exit potential | -10 V |

Table S5 - Pearson correlation matrix between essential elements in L. minor

| **A - Pearson Correlation Coefficients in *L. minor* Exposed to PFOA** | | | | | | | | |
| --- | --- | --- | --- | --- | --- | --- | --- | --- |
|  | | Na-P | K-P | Ca-P | Fe-P | Cu-P | Zn-P | Mo-P |
| Na-P | Pearson Correlation | 1 | -1.000^*^ | -.999^*^ | -1.000^*^ | -.973 | -.968 | -.999^*^ |
|  | Sig. (2-tailed) |  | .014 | .030 | .013 | .148 | .162 | .032 |
| K-P | Pearson Correlation | -1.000^*^ | 1 | .998^*^ | .999^*^ | .968 | .962 | .997^*^ |
|  | Sig. (2-tailed) | .014 |  | .043 | .027 | .162 | .176 | .046 |
| Ca-P | Pearson Correlation | -.999^*^ | .998^*^ | 1 | 1.000^*^ | .983 | .978 | 1.000^**^ |
|  | Sig. (2-tailed) | .030 | .043 |  | .016 | .119 | .133 | .003 |
| Fe-P | Pearson Correlation | -1.000^*^ | .999^*^ | 1.000^*^ | 1 | .978 | .973 | 1.000^*^ |
|  | Sig. (2-tailed) | .013 | .027 | .016 |  | .135 | .149 | .019 |
| Cu-P | Pearson Correlation | -.973 | .968 | .983 | .978 | 1 | 1.000^*^ | .983 |
|  | Sig. (2-tailed) | .148 | .162 | .119 | .135 |  | .014 | .116 |
| Zn-P | Pearson Correlation | -.968 | .962 | .978 | .973 | 1.000^*^ | 1 | .979 |
|  | Sig. (2-tailed) | .162 | .176 | .133 | .149 | .014 |  | .130 |
| Mo-P | Pearson Correlation | -.999^*^ | .997^*^ | 1.000^**^ | 1.000^*^ | .983 | .979 | 1 |
|  | Sig. (2-tailed) | .032 | .046 | .003 | .019 | .116 | .130 |  |
| *. Correlation is significant at the 0.05 level (2-tailed). | | | | | | | | |
| **. Correlation is significant at the 0.01 level (2-tailed). | | | | | | | | |

| **B - Pearson Correlation Coefficients in *L. minor* Grown in the Control Group** | | | | | | | | |
| --- | --- | --- | --- | --- | --- | --- | --- | --- |
|  | | Na-C | K-C | Ca-C | Fe-C | Cu-C | Zn-C | Mo-C |
| Na-C | Pearson Correlation | 1 | .811 | .999^*^ | .901 | .929 | .992 | .985 |
|  | Sig. (2-tailed) |  | .398 | .024 | .285 | .241 | .079 | .111 |
| K-C | Pearson Correlation | .811 | 1 | .833 | .984 | .970 | .877 | .900 |
|  | Sig. (2-tailed) | .398 |  | .374 | .113 | .157 | .319 | .287 |
| Ca-C | Pearson Correlation | .999^*^ | .833 | 1 | .917 | .943 | .996 | .991 |
|  | Sig. (2-tailed) | .024 | .374 |  | .261 | .216 | .054 | .087 |
| Fe-C | Pearson Correlation | .901 | .984 | .917 | 1 | .998^*^ | .948 | .963 |
|  | Sig. (2-tailed) | .285 | .113 | .261 |  | .044 | .206 | .174 |
| Cu-C | Pearson Correlation | .929 | .970 | .943 | .998^*^ | 1 | .968 | .979 |
|  | Sig. (2-tailed) | .241 | .157 | .216 | .044 |  | .162 | .129 |
| Zn-C | Pearson Correlation | .992 | .877 | .996 | .948 | .968 | 1 | .999^*^ |
|  | Sig. (2-tailed) | .079 | .319 | .054 | .206 | .162 |  | .033 |
| Mo-C | Pearson Correlation | .985 | .900 | .991 | .963 | .979 | .999^*^ | 1 |
|  | Sig. (2-tailed) | .111 | .287 | .087 | .174 | .129 | .033 |  |
| *. Correlation is significant at the 0.05 level (2-tailed). | | | | | | | | |
